# Low dose ionizing radiation effects on the immune system

**DOI:** 10.1016/j.envint.2020.106212

**Published:** 2020-12-05

**Authors:** Katalin Lumniczky, Nathalie Impens, Gemma Armengol, Serge Candéias, Alexandros G. Georgakilas, Sabine Hornhardt, Olga A. Martin, Franz Rödel, Dörthe Schaue

**Affiliations:** a National Public Health Centre, Department of Radiation Medicine, Budapest, Albert Florian u. 2-6, 1097, Hungary; b Belgian Nuclear Research Centre, Biosciences Expert Group, Boeretang 200, 2400 Mol, Belgium; c Unit of Biological Anthropology, Department of Animal Biology, Plant Biology and Ecology, Faculty of Biosciences, Universitat Autònoma de Barcelona, 08193-Bellaterra, Barcelona, Catalonia, Spain; d Université Grenoble-Alpes, CEA, CNRS, IRIG-LCBM, 38000 Grenoble, France; e DNA Damage Laboratory, Physics Department, School of Applied Mathematical and Physical Sciences, National Technical University of Athens (NTUA), Zografou 15780, Athens, Greece; f Federal Office for Radiation Protection (BfS), Ingolstaedter Landstr.1, 85764 Oberschleissheim, Germany; g Peter MacCallum Department of Oncology, The University of Melbourne, Melbourne 3052, Victoria, Australia; h Department of Radiotherapy and Oncology, University Hospital, Goethe University Frankfurt am Main, Theodor-Stern-Kai 7, 60590 Frankfurt am Main, Germany; i Department of Radiation Oncology, David Geffen School of Medicine, University of California at Los Angeles (UCLA), Los Angeles, CA 90095-1714, USA

**Keywords:** Low-dose ionizing radiation, Immune system, Epidemiological data, DNA damage response, Inflammation

## Abstract

Ionizing radiation interacts with the immune system in many ways with a multiplicity that mirrors the complexity of the immune system itself: namely the need to maintain a delicate balance between different compartments, cells and soluble factors that work collectively to protect, maintain, and restore tissue function in the face of severe challenges including radiation damage. The cytotoxic effects of high dose radiation are less relevant after low dose exposure, where subtle quantitative and functional effects predominate that may go unnoticed until late after exposure or after a second challenge reveals or exacerbates the effects. For example, low doses may permanently alter immune fitness and therefore accelerate immune senescence and pave the way for a wide spectrum of possible pathophysiological events, including early-onset of age-related degenerative disorders and cancer. By contrast, the so called low dose radiation therapy displays beneficial, anti-inflammatory and pain relieving properties in chronic inflammatory and degenerative diseases. In this review, epidemiological, clinical and experimental data regarding the effects of low-dose radiation on the homeostasis and functional integrity of immune cells will be discussed, as will be the role of immune-mediated mechanisms in the systemic manifestation of localized exposures such as inflammatory reactions. The central conclusion is that ionizing radiation fundamentally and durably reshapes the immune system. Further, the importance of discovery of immunological pathways for modifying radiation resilience amongst other research directions in this field is implied.

## 1. Introduction

**Fig. 1.**
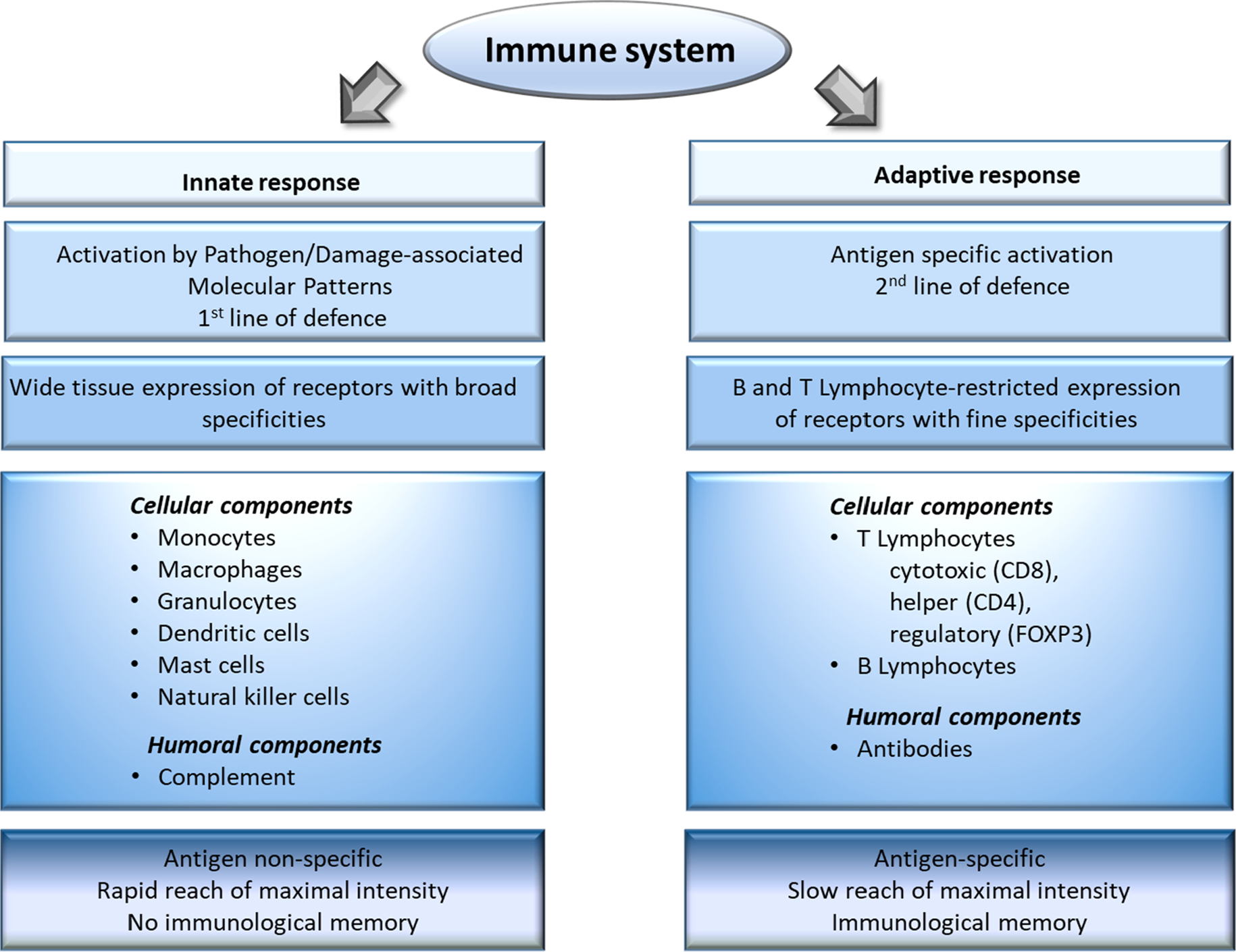
Schematic representation of the structure of the immune system and its major functional features. A molecule that is recognized by the immune system is called an antigen, which can be both self and non-self in origin. The immune system can be divided in two main compartments: the innate immune system and the adaptive immune system. The innate immune system is composed of a cellular compartment consisting of mononuclear cells (monocytes/macrophages, mast cells), polymorphonuclear cells (neutrophils, basophils, eosinophils), dendritic cells (DCs), innate immune cells (e.g. natural killer or NK cells) and the humoral complement system (Artis and Spits, 2015). Innate immune cells see danger through their germline-encoded pattern recognition receptors (PRRs), which recognize specific molecular structures present on pathogens (so-called pathogen-associated molecular patterns or PAMPs) or produced by damaged cells (so-called damage-associated molecular patterns or DAMPs) (Amarante-Mendes et al., 2018). Forming our first-line of defense, this recognition is relatively non-specific and quick, reaching its maximal intensity shortly after antigen encounter without yielding specific immunological memory. Phagocytosis is one of the main mechanisms for antigen elimination by innate immune cells. During danger recognition and antigen processing innate immune cells mature and release various soluble immune mediators called cytokines and chemokines, which drive inflammation and attract adaptive immune cells (Commins et al., 2010). In fact, an important role of the innate immune system is the activation of the adaptive arm. Macrophages and DCs in particular are professional antigen presenting cells with the unique ability to activate naïve cells of the adaptive immune system by displaying components of the processed antigens within the major histocompatibility complex (MHC) on their surface and present them to lymphocytes in the presence of necessary co-stimulatory signals (Wynn et al., 2013). Cells of the adaptive immune system include T lymphocytes (such as CD4 + helper, CD8 + cytotoxic and Foxp3 + regulatory) and B lymphocytes. T cells are responsible for cell-mediated immune response while B cells play role in humoral immune response (mediated by antibodies). In contrast to the innate immune system, the major features of the adaptive immune response are: high antigen specificity, latency of maximal response and development of immunological memory exemplified by faster and qualitatively different recall responses (Santana and Esquivel-Guadarrama, 2006). The first step in the activation of the adaptive immune system is antigen recognition by CD4 + or CD8 + cells through their highly antigen-specific T-cell receptors (TCRs). Professional antigen presenting cells present antigenic peptides conjugated either to MHCII, inducing CD4 + activation or to MHCI, contributing to CD8 + activation. Activated CD8 + T cells kill the antigen-presenting cells through the release of cytotoxic agents stored in intracellular granules, or directly by cell-to-cell contact engaging death receptors, or through the production of cytokines that trigger apoptosis. B cells, on the other hand, recognize extracellular antigens via their antigen-specific B cell receptor, which are essentially antibodies bound on the cell membrane forming a transmembrane receptor. Once activated with help from CD4 + T cells, B cells start to divide and differentiate into plasma cells which secrete huge numbers of soluble antibodies similar to the one that recognized the antigen in the first place (Hardy and Hayakawa, 2001). Circulating antibodies bind to their specific antigens and these antigen–antibody complexes induce activation of the complement system, which in turn leads to a rapid neutralisation by the proteolytic activity of the complement system and further phagocytosis by innate cells, i.e. antibody-dependent cellular cytotoxicity. Most of the intercellular communication in the immune system is guided through a complex system of chemokines, cytokines and interferons that affect trafficking, activation, differentiation and functional maturation (Turner et al., 2014). To prevent tissue damage from excessive immune activation multiple control mechanisms are in place that act through cell-to-cell contact or cytokines, involving among others regulatory T cells (Tregs) (Persa et al., 2015). Finally, to mount an effective response, immune components must circulate between the blood and lymph nodes, recognize antigens upon contact with presenting cells, and differentiate to effector T cells and plasma cells. Moreover, these cells must extravasate the lymph nodes, migrate to affected tissue to secure host-protective activities and to recircle to blood to counteract chronic activation (Germain et al., 2012). Accordingly, one has to consider a high degree of cellular motility and interaction dynamics of the immune system.

The immune system is the body’s main defence mechanism able to distinguish between self and non-self as well as sensing danger. Its main function is to recognize and eliminate different pathogens and damaged or abnormal cells within the body (Fig. 1) (Murphy and Weaver, 2017).

Direct ionizing radiation (IR) effects on the immune system are well-documented and were among the first radiobiological observations made soon after the discovery of X-rays (Anderson and Warner, 1976; Radiation, 1972; Schaue, 2017). With the discovery of antibiotics and anti-inflammatory drugs, together with an increased awareness of radiation carcinogenesis the initial enthusiasm to treat infections and benign diseases with radiation subsided for the most part and was replaced with a sole focus on cancer radiotherapy (RT) using doses that can kill cancer cells (i.e. high doses delivered in multiple fractions, generally 2 Gy per daily fraction). As a result, the radiation literature tends to be dominated for the most part by high dose exposure studies with single doses above 1 Gy (Schaue, 2017). Data from radiation oncology patients come with generally well-defined dosimetry and clinical monitoring, but the presence of a tumour is an important confounding factor from an immunological point of view. For instance, many patients receive fractionated local tumor irradiation of up to 74 Gy total dose as part of an extensive combined modality treatment that can include chemotherapy, surgery and/or immunotherapy (Demaria et al., 2015). Therefore immunological alterations during cancer treatment may not necessarily be due to radiation effects alone. Studies based on high dose IR applied to cancer patients indicated that radiation had immune suppressive properties and this paradigm prevailed in the scientific literature for decades (Anderson and Warner, 1976; Balogh et al., 2013; Hader et al., 2020; Kachikwu et al., 2011; Lumniczky and Safrany, 2015; McFarland et al., 2012; Merrick et al., 2005). On the other hand low (below 100 mGy) and intermediate dose (between 100 mGy and 1 Gy) exposure scenarios are much more relevant for the general population as they may have public health consequences. They are also much more difficult to study because toxicity and carcinogenesis are a lot less obvious while other, more subtle functional alterations gain in importance. DNA damage correlates with the dose and the probability of severe direct DNA damage after low doses and dose rates is low, thus other mechanisms may prevail (Mothersill and Seymour, 2014). Within the long time span between exposure and the onset of the clinically apparent pathologies, likely persisting alterations in the functional integrity of the organism as a whole must be present leading to the development of a pathology. Not only the DNA-damage response (DDR) related pathways, but also other, immune-related pathways may contribute to both cancer and non-cancer health outcomes from exposure to IR. Hence, a holistic approach that integrates these multiple mechanisms at all organizational levels is needed to understand the complex response system (Mavragani et al., 2016).

An accumulating amount of scientific evidence based on epidemiological and pre-clinical studies indicate that low dose exposures might directly impact immune functions and - although controversial -, these data indicate that IR may not only be immune suppressive (Cui et al., 2017; Hellweg, 2015; Makinodan and James, 1990; Rodel et al., 2015; Sambani et al., 1996; Xu et al., 1996). A comprehensive review of available data on IR effects on the immune system was published in the UNSCEAR 2006 report (Radiation, 2008) including both high and low dose effects and highlighted complex functional changes within the immune system in response to radiation. This was the first report released by an international organisation investigating radiation health effects which abandoned the “classical” paradigm that IR is purely immune suppressive. Actually, this report proposes to consider IR as an immunomodulatory agent due to the multitude and sometimes opposing ways it can influence the immune system depending on various parameters such as dose, dose rate, genetic background, age, health status, comorbidities, lifestyle, environmental co-stressors, etc (Radiation, 2008).

This review is part of a collection of papers summarizing discussions of the MELODI workshop on non-cancer effects of low dose IR, organized in Sitges, Spain, 10–12 April 2019. Here we aim to provide an update on low dose IR effects on the human immune system with the goal to summarize what is “known”, what is suspected but still controversial and what is “not yet known” based on existing epidemiological, clinical and pre-clinical data (Table 1).

## 2. Human biomonitoring and epidemiological data on low dose radiation-induced immunological changes

Data from genuine epidemiological studies on immune alterations in the context of low dose exposures remain scarce (Table 2). This may be because symptoms of immune-related diseases do not appear in a form or along a time course that can easily be related to radiation exposure apart from the well-known high radiation sensitivity of many resting lymphocytes.

A substantial amount of data is available from different mass casualties implying acute or chronic exposure scenarios. Among the best studied cohorts today are A-bomb survivors and clean-up workers (liquidators) of the Chernobyl nuclear power plant accident, subjected to single dose acute exposure.

### 2.1. A-bomb survivors and Chernobyl clean-up workers cohorts

Data regarding radiation effects on the immune system of A-bomb survivors started to accumulate in the 1980’s and have been periodically updated ever since. The caveat is of course that there may be influences of race and life style factors. Akiyama reported from the Life Span Study on about 120,000 people who had been exposed to an average of 0.16 Gy according to the dosimetry system revised in 1986 (DS86), which includes “non-exposed” controls (Akiyama, 1995; Akiyama et al., 1983). Many long-term immune effects were observed that generally suggested a shift in the peripheral lymphocyte balance in favor of B cells with increased serum immunoglobulin (Ig) levels at the expense of cluster of differentiation 3 (CD3) + T cells which were reduced in both numbers and functionality. A dose-dependent drop in naïve CD4+ and CD8+ T lymphocytes was recorded, while memory cells were less affected (Kusunoki et al., 1998; Kusunoki et al., 2003). That T cell differentiation and development may be permanently altered was suggested by a rise in rare double negative CD4−CD8−, alpha/beta T cells (Kusunoki et al., 2003; Kyoizumi et al., 2010). The appearance and persistence of TCR-mutant T cells was detected mostly in the memory CD4+ T cell compartment in a dose-dependent manner in individuals aged 20 or older at the time of bombing (Kusunoki et al., 2003). A relatively robust dose-dependent readout for TCR mutant frequency was suggested after studies on Chernobyl clean-up workers, even at doses of 0.25 Gy (Saenko et al., 2000). This would suggest the use of TCR mutation as a potential biodosimeter relevant to T cell function.

In many ways, immune changes observed in A-bomb survivors resemble those associated with aging. It was reported that the output of naïve T cells was reduced, the memory T cell pool was expanded while the TCR repertoire became limited, all of which were associated with low grade inflammation that involved myeloid cells known as inflammaging (Denkinger et al., 2015; Franceschi et al., 2019; Fulop et al., 2018; Kusunoki and Hayashi, 2008; Kusunoki et al., 2010). Doses in the range of 0.005–0.2 Gy drove accelerated thymic involution, which could still be evident 30 years later when the natural, age-related process was well under way if not completed (Ito et al., 2017). The frequency and counts of monocytes were dose-dependently increased by radiation exposure and this increase was more pronounced after 60 years showing a possible acceleration of age-dependent clonal haematopoiesis (Yoshida et al., 2019). However, the response to vaccination in elderly atomic bomb survivors seemed not to be impaired by radiation exposure early in life (Hayashi et al., 2018).

The role of IR in promoting accelerated aging was further demonstrated by looking at telomere length in leukocytes of A-bomb survivors. Lustig et al. showed that circulating leukocytes in A-bomb survivors had shorter than expected telomeres, and impaired function, which was dependent on dose and age at exposure. It was more severe in the young than in the elderly, showing a significant dose-dependency in individuals younger than 12-years at exposure. The authors concluded that because this was measurable in the progeny decades later, the initial lesion was the likely source. The association of telomere shortening with circulating biomarkers for aging, such as cytokine production and peripheral blood cell counts were lost in irradiated individuals indicating that radiation effects override those of aging. The authors hypothesized that the resultant functional defects may not necessarily be a disadvantage as myeloid cells may produce fewer inflammatory cytokines, whereas this argument is difficult to make for reduced T cell function (Lustig et al., 2016). Of special interest to the discussion here might be the observation by Yoshida el al. who showed a biphasic alteration of CD4+ telomere length with irradiation dose: longer telomeres after low dose exposure and progressively decreasing telomere length with doses above 0.5 Gy which correlated with the individual metabolic status (Yoshida et al., 2016).

Further low-dose IR induced quantitative and functional alterations in immune parameters in Chernobyl clean-up workers were reported by Ilienko et al., who investigated cellular immune parameters of 235 individuals exposed to doses between 0.1 and 3500 mSv. They found decreased CD4+/CD8+ ratios and increased Tregs in 56% of the studied clean-up workers, especially in those exposed to low doses. Interestingly, the authors showed that the decrease in the level of B cells and activated T cells correlated with the increase in interleukin (IL)-1β levels in individuals exposed to doses below 100 mSv (Ilienko et al., 2018). Oradovskaia et al. studied a cohort of clean-up workers who developed different malignant diseases and identified typical immune parameter changes in these people 1–3 years before the manifestation of cancer. These changes include reduced CD3+ CD4+ T cell levels, increased CD8+ T cell levels and hence a reduced CD4/CD8 ratio, and a prevalence for natural killer T (NKT) cells over conventional natural killer (NK) cells (Oradovskaia et al., 2011b) confirming previous data of increased NKT cells in clean-up workers (Kuzmenok et al., 2003). Though these markers seem to indicate a risk for cancer development, it is not clear to what extent these alterations were specific for radiation exposed individuals rather than the general population developing cancer at later times.

### 2.2. Environmental or occupational radiation exposures

Other exposure scenarios of interest are long-term chronic exposures affecting either residents living in regions with increased background radiation (natural or human-made) or workers occupationally exposed to radiation. Studies investigating individuals living in Ramsar, Iran at natural high background radioactivity of up to 260 mSv per year showed mild immunological alterations manifested in increased Ig levels (in particular IgE), increased levels of activated CD4+ cells and a tendency towards T helper 2 (Th2) polarization without changes in innate immune parameters (in terms of neutrophil chemotaxis), while results relating to cytogenetic damage in blood lymphocytes were contradictory (Attar et al., 2007; Borzoueisileh et al., 2013; Ghiassi-nejad et al., 2002; Ghiassi-Nejad et al., 2004; Molaie et al., 2012). Studies investigating residents living in the Yangjian high background radiation area (with cumulative doses up to 249 mSv) showed an increased tendency of both CD4+ and CD8+ T cells in the peripheral blood mononuclear cells (PBMCs), the latter correlating with dose. Moreover, multiple inflammation-related cytokines and blood proteins were significantly increased, such as soluble IL-6 receptor (sIL-6R), interferon (IFN)-γ, monocyte chemoattractant protein-1 (MCP-1) and C-reactive protein (CRP) (Li et al., 2019). Chang et al investigated home environments giving off on average 169 mSv gamma radiation from building materials to 196 individuals over 2–13 years. They found a change in lymphocyte subsets in favour of CD8+ T cells while CD4+ T cell numbers and CD4/CD8 ratio negatively correlated with dose when compared to 55 non-exposed close relatives (Chang et al., 1999a). Despite the fact that the cited studies focused on different immune parameters their conclusions were quite convergent, showing increased activity in the adaptive immune response and the presence of pro-inflammatory factors. A recent study on gene expression profiles of PBMCs from inhabitants of the high level background radioactivity area in Kerala, India found that immune response pathways were among the radiation-affected over-represented pathways (Jain and Das, 2017).

Immune markers were investigated in people living in radiation-contaminated areas such as around the Mayak nuclear complex, including the Techa River in the Russian Federation. Akleyev et al. showed that the innate immune system, NKT cells, and neutrophils in particular, might be activated by chronic exposure to approx. 0.9 Gy in a dose and dose-rate dependent manner in residents of Techa River (Akleyev et al., 2019). Another study indicated a dose-dependent decrease in the concentration of T helper cells, reduced IFN-γ levels, increased NKT lymphocyte numbers as well as transforming growth factor beta (TGF-β), matrix metalloproteinase 9 (MMP-9), IgA and IgM levels in Mayak workers exposed to external gamma rays with or without internal alpha radiation. Authors concluded that changes detected in the immune parameters of the investigated individuals favoured the maintenance of a chronic inflammatory status, which could contribute to the development of radiation-related late pathologies such as cardiovascular and malignant diseases (Rybkina et al., 2014). Very similar conclusions were reached by Kiselev et al., who reviewed immunological changes in radiation workers at a Siberian chemical complex (over 4000 workers), at the Mayak nuclear facility and at the Chepetsk chemical and metallurgical plant. The common characteristic of these facilities was the presence of a mixed-type exposure (external gamma rays and internal alpha radiation due to incorporated uranium). Importantly, the study recorded not only basic laboratory changes in immune parameters but also clinically relevant symptomatic immune dysfunctions such as infections, allergies, autoimmunity and immunoproliferative diseases and found an increased risk for immune deficiency leading to an infectious syndrome in nuclear industry workers compared to controls. Significantly elevated IgE levels in a certain group of workers in the absence of a relevant allergic anamnesis was also reported, which might indicate a low dose radiation induced imbalance in humoral immunity (Kiselev et al., 2017). Gyuleva et al. basically confirmed most of these findings in Bulgarian nuclear power plant workers with additional details on individual lymphocyte subpopulations and their activation status. Namely, they showed a subtle but significant decrease in the proportion of naïve CD4+62L+ cells, CD4+CD25+ activated/regulator T cells and an increase in activated CD8+CD28+ cytotoxic T cells, along with an increase in NKT cells in persons receiving doses below 200 mSv. Thus, they hypothesised a possible shift from a Th1 to Th2 response at doses above 200 mSv. The main added value of this study compared to the previous ones was that a) it took into account confounding factors such as smoking and alcohol consumption and b) it discussed the influence of aging as another confounding factor on immune parameters, which can overlap with certain changes seen following radiation. Though, they also showed that most of the changes in the measured parameters remained within the normal reference values illustrating the discrete effects of low doses (Gyuleva et al., 2018; Gyuleva et al., 2015a; Gyuleva et al., 2015b). Other occupational data are from interventional cardiologists, radiologists and radiation workers. These studies generally report exposure doses well below those found in nuclear industry workers mentioned above. At doses of 8.14 ± 7.81 mSv/year for at least 5 years, i.e. below 50 mSv in cumulative dose, drastic fluctuations in circulating immune cells are unlikely to occur, however more subtle changes such as priming the immune system towards stronger Th1 response upon a secondary challenge were reported (Ahmad et al., 2016; Karimi et al., 2017; Zakeri et al., 2010). Occasional quantitative changes in the cellular and humoral immune system components have been reported even at standard annual dose levels below 3.5 mSv/year with smoking being an important confounder (Godekmerdan et al., 2004; Klucinski et al., 2014; Rees et al., 2004). High variability amongst “normal” individuals is an important limitation, especially as pre-exposure levels are rarely if ever known in immune epidemiological studies. Diurnal rhythm has a pronounced effect that is impossible to control and individual variation in lymphocyte radiosensitivity is also large.

### 2.3. Studies on radiation-exposed children

There is a limited number of studies investigating immune changes in children exposed to chronic low dose exposures. In general, as reviewed in the UNSCEAR 2013 report, children might be more at risk for a number of radiation-induced late effects both of stochastic and deterministic nature. This is true for the incidence of certain tumors such as leukaemias, skin, brain and thyroid cancer as well as deterministic health effects such as cognitive defects, cataracts and thyroid nodules (Radiation, 2014). For this reason and also for the fact that the immune system maturation in children is still ongoing and damage at this state might lead to different consequences in immune function than in adults or older individuals with various degrees of immune senescence, we considered discussing studies of radiation-exposed children separately from those targeting adults. Chronic low dose exposure in children of kindergarden-age can have long-lasting effects on peripheral blood cell counts. Children exposed to 21–85 mSv gamma radiation over a 1–2 year period experienced a drop in total leukocyte and neutrophil counts and an increase in eosinophils that lasted as long as 5–7 years after the end of exposure, with total lymphocyte numbers not affected when compared to children exposed to 2–5 mSv (Chang et al., 1999). Alterations in T cell immunity were detected in children 6–13 year old living within a 40–75 km radius of radiation-contaminated areas around Chernobyl in North Ukraine and exposed to persistent low level radiation. These children had lower CD4 + T cell counts and a shift in the immune balance towards cytotoxic T cell and NK cell subsets especially in vulnerable populations such as those who developed recurrent respiratory problems (Vykhovanets et al., 2000). The effects were more pronounced at doses above 1 mSv but were also seen below that dose (Chernyshov et al., 1997), although confounding factors such as general health status, individual immunogenetic make-up and exposure to pathogens make radiation dose response patterns difficult to ascertain. Though in children with irritable bowel disease living in the Chernobyl contaminated area no association with radiation was found (Sheikh Sajjadieh et al., 2012).

The link between childhood radiation exposure and thyroid cancer was firmly established in the aftermath of the Chernobyl accident in 1986 (Pacini et al., 1997), with non-cancer thyroid diseases including autoimmune thyroid disorders emerging a few years after the accident. This was less of a research focus in the A-bomb survivor cohorts, although thyroid pathologies were studied in middle aged adults from the Hiroshima and Nagasaki cohorts who were exposed in utero, with a mean maternal uterine dose of 0.256 Gy (range 0.005 Gy to 1 Gy). It was determined that they had similar risk estimates as those in the Chernobyl study but there was no significant linear dose response for thyroid nodules or autoimmune thyroid disease (Imaizumi et al., 2008). Similarly, thyroid nodules tended to be much more prevalent in the radiation-exposed Marshallese population, but without correlating to abnormal thyroid function (Takahashi et al., 1999). A comprehensive review on the initial epidemiological studies is provided by Saenko et al., who concluded that the frequencies of abnormal haematological parameters and thyroid autoimmunity did not correlate with dose (Saenko et al., 2011). According to long-term studies, the rise in autoantibodies is very common even at lower doses but it can be transient and does not necessarily yield clinical thyroid autoimmune disease or thyroid dysfunction although well-designed long-term investigations are still needed (Agate et al., 2008; Eheman et al., 2003; Kasatkina et al., 1997; Ron and Brenner, 2010). The disconnection between the rise in autoantibodies and the lack of clinical disease might be due to a radiation damage-driven release of thyroid antigen without subsequent lymphocytic infiltration. One explanation is the possibility of a transient autoimmune reaction (Agate et al., 2008). It is also important to consider the dietary iodine uptake. However, determining the association between low dose exposures and their clinical significance in terms of benign thyroid diseases is not without challenges, which include limited sample size, inadequate dose estimates, prevalence in healthy subsets, gender differences and technical problems that relate to the variety of antibodies that can be studied (Eheman et al., 2003).

Overall, epidemiological studies on long-term low dose irradiation effects on the immune system indicate a) a consensus regarding persistent alterations in CD4+ T cell numbers and function; b) a shift towards humoral immunity; c) contradictory conclusions on cytotoxic CD8+ T cell numbers and innate immunity, most probably with a shift towards activation of certain NK cell compartments; d) limited knowledge on changes in granulocytes; e) likely accelerated immune aging (Table 3). Overall, there are big gaps in our understanding of functional alterations in immune parameters which are of particular interest at this dose range.

Results from the diverse epidemiological or human biomonitoring studies are difficult to compare mainly due to gross heterogeneities in the studied cohorts in terms of age (both in terms of age at exposure and time elapsed from exposure), doses received, irradiation scenarios (acute or chronic, external, internal or mixed exposures), presence of confounding factors (lifestyle, comorbidities, genetic background) and studied endpoints. A major drawback to almost all of these studies is their purely descriptive nature without much effort to link these to specific pathological conditions or diseases. Studies investigating immune alterations in A-bomb survivors suggested a correlation between radiation-induced chronic inflammation and increased incidence of chronic degenerative-type conditions (e.g. cardiovascular diseases, metabolic alterations). The few observations pointing to an increase in the incidence or susceptibility to infections or towards an increased predisposition for autoimmune disorders await confirmation by further studies. So far, it has been difficult to discriminate between immune alterations that are within the normal resilience capacity of an individual versus those that fall outside that range and might link directly to certain diseases. The fact that immune system alterations are part of many if not all pathophysiological processes leading to chronic diseases not traditionally considered as bona fide immune diseases (e.g. heart conditions) only adds further to the complexity of the issue. A meta-analysis of all epidemiological and biomonitoring studies would be useful to evaluate relevant correlations between radiation effects, immune system changes and related health consequences. A better characterisation of the control group and the study design (including the statistical analysis used), categorisation of the measured endpoints and reported health outcomes would help identifying the most informative studies.

## 3. Experimental and pre-clinical data on low dose ionizing radiation effects on the immune system

The number of *in vitro* and *in vivo* data about low dose IR effects on the immune system is increasing in parallel with a progressively increasing concern of the medical and scientific community regarding long-term biological effects of low doses. These experimental evidences often confirm epidemiological observations and shed light on the mechanisms how IR interacts with the immune system. In the following paragraphs, we will review the most pertinent experimental findings which complement epidemiological data, focusing on the link between basic molecular mechanisms targeted by IR and the immune system, as well as direct low dose effects on quantitative and functional changes in the different immune compartments. Finally, we will present regulation of the inflammatory response as an example of the differential mechanisms and outputs initiated by low-dose versus high-dose irradiation.

### 3.1. Association between DNA damage response and immune response at low doses of radiation

DNA damage is considered a primary consequence of IR and the activation of the DDR pathway is a key factor in determining long-term cell fate after irradiation. IR at low doses already may induce a variety of lesions like double-strand beaks (DSBs) accompanied by single-strand breaks (SSBs) and/or oxidized bases in a bistranded or unistranded form. Manning et al showed increased frequency of micronucleated erythrocytes in the blood of mice treated *in vivo* with either external low dose X rays or with PET scan associated internal exposure with the radioisotope ^18^F-FDG (Manning et al., 2014). Rothkamm et al reported that quantification of γH2AX foci in the leukocytes of patients subjected to CT scans could reliably estimate the level of radiation exposure (Rothkamm et al., 2007). DNA damage after such low doses is not sufficiently severe to induce cells death but can initiate danger signalling (Mavragani et al., 2016; Mavragani et al., 2017). Interestingly, both *in vitro* and *in vivo* studies have shown a remarkable persistence of the DSBs (as evidenced by the slow resolution kinetics of γH2AX foci) even after doses in the range of computed tomography (CT) scans, well below 100 mGy. These changes were first detected and extensively studied in fibroblasts (Grudzenski et al., 2010; Lobrich et al., 2005; Rothkamm and Lobrich, 2003) but actually more recent investigations showed a similar persistence and delayed repair kinetics of DNA damage in lymphocytes as well (Beels et al., 2010; Lassmann et al., 2010). Plenty of evidence suggests a strong crosstalk between DDR activation and inflammatory response triggering primarily innate immune responses (Ermolaeva and Schumacher, 2013; Pateras et al., 2015) also in case of deficient DNA repair or persistent DNA damage (Karakasilioti et al., 2013). Damaged cells can release a variety of stress or danger signals called damage associated molecular patterns (DAMPs) which act as mediators of innate immune responses (Heil and Land, 2014). These endogenous molecules released from damaged, stressed or dying cells, in analogy to the pathogen-associated molecular patterns - are recognised by the pattern recognition receptors located within and on innate immune system cells and instigate inflammatory responses. DAMPs can be different cytokines, DNA, RNA, ATP, intra-cellular proteins or protein fragments, etc. It is remarkable, that this feature of stress response is common with variations across organisms of different complexity and evolutionary phylogeny i.e. from plants to mammals (Land, 2015; Pavlopoulou et al., 2019).

### 3.2. Systemic and abscopal effects of local radiotherapy

Although typical fraction doses for RT are 2 Gy to the tumor, neighboring tissues can receive a wide range of low to medium doses up to 0.5 Gy (Pouget et al., 2018). In many cases of RT, biological and clinical systemic immune and inflammatory responses were found to be similar to responses of the irradiated tissues reflected in changes of key modulators of the immune system like cytokines or chemokines. For example, in a study by Mathias et al differential anti- and pro-inflammatory responses were detected in the heart after local heart irradiation with low and high doses and these differential responses were partly reflected at systemic level, in the plasma as well (Mathias et al., 2015). Several studies in human patients undergoing RT support a time- and treatment-dependent modulation of specific cytokines at systemic level, which in some cases can persist up to several years post-treatment, indicating a definite systemic response to radiation mediated by inflammatory pathways (Marconi et al., 2019). These systemic phenomena have also been observed in animals as reviewed in (Mavragani et al., 2017). Of course, these excessive immune responses not only contribute to chronic inflammation and tissue damage but also to clearance of damaged cells and tissue remodeling/regeneration (Ermolaeva and Schumacher, 2014).

Thus, local RT can cause changes in tissues and organs outside the field of irradiation. This is called the abscopal effect (Siva et al., 2015) where it can cause damage to unirradiated normal tissues, or to a distant, non-irradiated tumor. Clinically, such effects are well recognised, as patients often suffer from fatigue, diarrhea and weight loss during local RT. Reports of spontaneous anti-tumour abscopal responses are rare, since an immune tolerant state has already been established in the patient; Abuodeh et al reported 35 cases over 45 years, out of millions of RT patients treated during this period (Abuodeh et al., 2016). Since the first report by Demaria et al that the immune system was an integral component of the abscopal response (Demaria et al., 2004), there have been studies reported, in which activation of the immune system, commonly using checkpoint inhibitors against cytotoxic T lymphocyte antigen 4 (CTLA-4), programmed cell death protein 1 (PD-1) and OX40 in combination with local RT, was able to induce growth suppression in the second unirradiated primary tumor (Esposito et al., 2015). On the other hand, under optimal conditions (eg targeting a hypoxic core of the tumour with ablative RT), the anti-tumor effect can be achieved with RT alone (Tubin et al., 2019). Exposure of blood and normal tissues surrounding the tumor, including the bone marrow and thymus, to low-dose scatter radiation, may play a role in the abscopal effects affecting normal non-irradiated tissues. After both external beam- and radionuclide RT, persistent and statistically significant increased DNA damage was observed in circulating PBMCs, and their repair capacity was affected (Denoyer et al., 2015; Yin et al., 2019). This could be explained by protracted induction of DNA damage by abscopal effects in susceptible progenitor cells that is revealed later as increased unrepaired damage in PMBCs.

### 3.3. Low dose radiation induced quantitative and functional changes in immune parameters

Lymphocytes are very sensitive to high dose irradiation. Though, a more in-depth analysis reveals significant differences in the radiosensitivity of the different immune cell subsets even after high doses with B and CD8+ T lymphocytes being more radiosensitive while dendritic cells (DCs), monocytes, macrophages and regulatory T cells being more radioresistant (Heylmann et al., 2014). Radiosensitivity of NK cells is controversial, with some studies reporting that NK cells are more radioresistant than CD3+ cells (Bogdandi et al., 2010; Swanson et al., 2020), while others reporting the opposite (Falcke et al., 2018).

Low dose radiation elicits a more heterogeneous immune response to radiation. Using murine models, it was shown that single low doses (up to 100 mGy) induced mild and transient (up to 21 days post irradiation) decrease in the pool of all the above mentioned splenic subpopulations (Bogdandi et al., 2010; Gridley et al., 2009). If similar doses were delivered chronically over a period of 1–17 weeks, no changes were detected in the short-term (Shin et al., 2010) but a long-lasting increase in T and B cell numbers were registered in mice after antigen stimulation (Ina and Sakai, 2005). Much lower doses (100 mGy/year) on the other hand did not impact on T or B cell pools, indicating these doses were too low to induce immune effects (Courtade et al., 2001). TCR repertoire profiling in mice showed that acute low/intermediate exposure could promote premature immune aging called immunosenescence. Exposure to a single dose of radiation was found to accelerate ageing of the peripheral TCR repertoire. The consequences of exposure were already visible after 1 month and lasted for 6 months. Interestingly, they were more pronounced in animals exposed to 0.1 Gy than in those exposed to 1 Gy, where they were partially corrected with time, indicating that they can be reversed/attenuated. Of note, some of these effects were due to radiation-induced impairment of hematopoietic stem cells (Candeias et al., 2017).

Chronic low dose rate irradiation of mice induced a) stimulation of innate immunity by enhancing the cytotoxicity of pre-stimulated NK cells (Sonn et al., 2012; Yang et al., 2014), b) myeloid cell differentiation and activation, c) suppression of pro-inflammatory responses (Shin et al., 2010) and d) a shift towards a Th2-type T cell phenotype (Shin et al., 2010) due in part to radiation-induced gene expression alterations in CD4+ T cells (Cho et al., 2018). Low dose effects on DCs are less well characterized. Persa et al. showed that low and high dose acute irradiation induced qualitatively different functional changes in murine splenic DCs *in vivo*. By this, low doses stimulated antigen uptake and lowered antigen presentation while high doses did not influence these parameters, on the other hand high doses increased the expression of T cell costimulatory markers and enhanced the production of pro-inflammatory cytokines (Persa et al., 2018). Other groups performing *in vitro* irradiation with doses ranging from 0.05 to 5 Gy of DC-precursors or mature DCs stimulated by lipopolysaccharide (LPS) reported no influence of irradiation on surface marker (CD80, CD83, CD86), cytokine expression or the capacity of the DCs to stimulate T-cell proliferation (Jahns et al., 2011). Moreover, a co-incubation of bone marrow-derived DCs with supernatant of LPS stimulated and irradiated macrophages resulted in a diminished CD40 expression but did not impact on the DC-derived induction of T-cell proliferation (Wunderlich et al., 2019).

Toll-like receptors (TLR), appearing on innate immune cells induced DC maturation which primed T-cells. In the presence of costimulatory elements, cytokine release was established which lead to macrophage activation (Ratikan et al., 2015). Low dose fractionated irradiation of mice lead to increased phagocytic activity of macrophages and increased CD8+ T cell cytotoxicity (Pandey et al., 2005). Another pathway important in radiation response from the immune system is through nucleotide binding oligomerisation domain (NOD)-like receptors. These are inflammasomes, which are important in case of damage from IR or from viruses. The redox status of cells may work as a rheostat, which may lead to a vicious circle of harm in case of excess oxidation, or may calm down the inflammation in reduced conditions. TLR agonists like LPS (especially for TLR4), may influence the response to ionizing radiation (Ratikan et al., 2015).

As previously mentioned, RT can induce long lasting systemic changes in immune/inflammatory parameters (Marconi et al., 2019). These changes may appear gradually during the course of the treatment (Manning et al., 2017), and even further evolve in the weeks following its completion (Balazs et al., 2019; Frey et al., 2020). Hence, the modulation of the expression of some inflammatory (*ARG1, BCL2L1, MYC*) genes in blood cells was not apparent after the first two fractions but became significant after the 25th in patients treated for endometrial cancer. Of note, RT did not modulate these same genes as strongly in head and neck patients (Manning et al., 2017). This difference may be due to different volumes of blood being exposed at each fraction in endometrial and head and neck patients, or to different effects of the different tumour entities on the immune system. The presence of a carcinoma in the head and neck area have indeed been shown to promote an immunosuppressive state in patients, which is even accentuated (increase in Tregs, increased expression of checkpoint molecules on CD4+ T cells), during and after RT (Balazs et al., 2019). Systemic modulation of cellular and humoral immune parameters can also depend on the mode of radiation delivery (Frey et al., 2020). However, most of these studies addressed changes occurring after repeated localized exposure, and it is therefore difficult to assess the exact role of low dose in these observations. Radiation-induced gene expression changes were also investigated in peripheral blood cells of RT-treated prostate cancer patients (El-Saghire et al., 2014) 18–24 hours after the first fraction (2.09 Gy), resulting in an equivalent total body blood dose of around 31 mGy. Gene set enrichment analysis showed the activation of several pathways involved in immune signaling: IFN secretion, CD28 stimulation, antigen processing and presentation, B-cell receptor (BCR) and TCR activation, inflammasomes, transcription factor nuclear factor kappa B (NF-κB), TLR and cytokine signalling indicating that local high dose resulted in systemic activation of pathways involved in immune processes. Interestingly, the comparison of the response elicited in whole human blood exposed *in vitro* to 0.05 or 1 Gy suggested that low dose radiation rather induced pro-survival/anti-apoptotic and immune response pathways, whereas high dose exposure rather induced p53-dependent signaling pathways (El-Saghire et al., 2013b). A similar dichotomy was found for the response to low and high dose radiation of purified human monocytes exposed *in vitro* (El-Saghire et al., 2013a). Low, but not high dose exposure induced the transcription of genes involved in TLR signaling, with a concomitant activation of NF-κB, p38, extracellular-signal-regulated kinase (ERK) and c-Jun N-terminal kinase (JNK). In contrast, exposure to 1 Gy induced phosphorylation of p53 and transcription of p53-dependent genes (El-Saghire et al., 2013b). *In vitro* exposure of human PBMCs obtained from healthy donors to 0.05 Gy of X rays or ^12^C ions did not induce significant changes in the proportion of T or NK cells 24 h later. Both types of radiation induced the transcription and the secretion of IL-2, IFN-γ and TNF-α, and an increase in the cytotoxic activity of the irradiated PBMCs. These effects appeared stronger with high than with low LET radiation (Chen et al., 2010).

Altogether, experimental data do not indicate a linear dose response relationship of immunological parameters modulation. IR may have qualitatively different effects at different doses/dose ranges. However, some responses/endpoints display discontinuous characteristics shared with non (DNA)-targeted properties of IR (Mothersill and Seymour, 2011). The molecular mechanisms responsible for these non-linear dose response relationships, however, remain elusive and may be associated with a connexion of multiple pathways initiated at different threshold doses and following different kinetics (Mothersill and Seymour, 2011; Rodel et al., 2012). There is growing evidence for a mechanistic link between transcription factor activity including p53 protein, activating protein 1 (AP-1), and NF-κB in the regulation of the DDR and that of immune response (Habraken and Piette, 2006; Magne et al., 2006; Prasad et al., 1995). For example, a dose-dependent biphasic transcriptional activity of NF-κB has been shown in endothelial cells (ECs) and macrophages (Rodel et al., 2004; Wunderlich et al., 2015).

### 3.4. Initiation of anti-inflammatory vs pro-inflammatory processes by IR

**Fig. 2.**
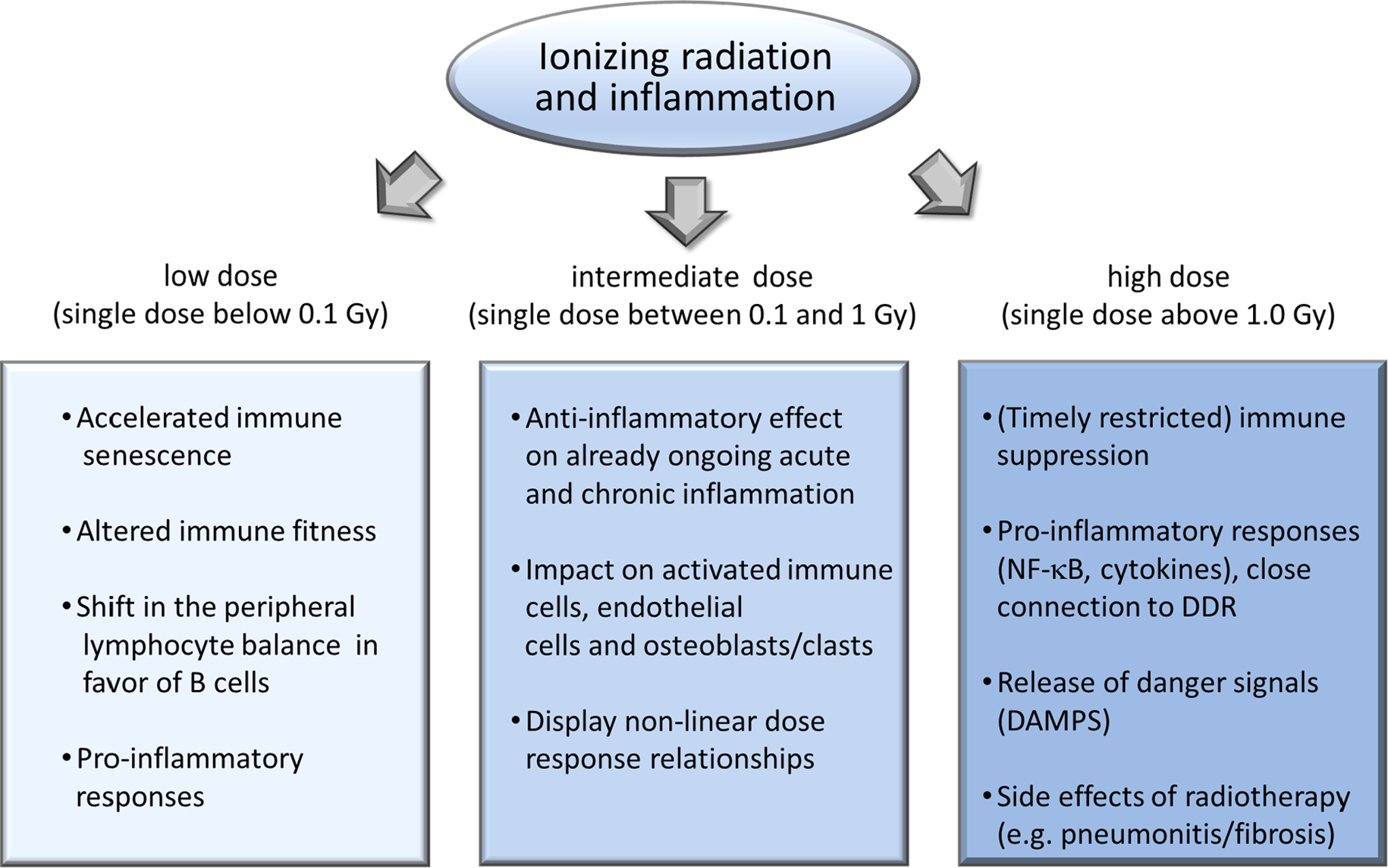
Schematic representation of the most important immune- and inflammation-related processes developing after low, intermediate and high dose irradiation based on available epidemiological, clinical and experimental data.

Functional changes in immune parameters after low dose exposure show a significant degree of heterogeneity and are often qualitatively different from changes induced by high doses. Inflammatory response is the best studied immune mechanism which is regulated differentially by low/medium dose and high dose irradiation. In the next paragraphs we summarize the current knowledge on the regulation of inflammatory processes by low versus high dose irradiation (Fig. 2).

#### 3.4.1. Modulation of basic inflammatory mechanisms by low/medium dose radiation exposure

Inflammation is a basic immunological effector process in response to harmful stimuli, such as pathogens, damaged cells or irritants (Murphy and Weaver, 2017) and is regulated by complex interactions between a variety of immune components and soluble mediators.

Among the initial step in the inflammatory pathways is the recruitment of leukocytes to the site of damaged tissue mediated by local adhesion to the endothelium. By analysing PBMC and polymorphonuclear (PMN: granulocytes) cell attachment to cytokine TNF-α activated endothelium a significant reduction of adhesion in the dose range of 0.3–0.7 Gy was among the first mechanisms reported to contribute to an anti-inflammatory effects of intermediate dose exposure (Hildebrandt et al., 2002; Kern et al., 2000; Roedel et al., 2002). These findings functionally coincide with secretion of the anti-inflammatory cytokine TGF-β1 from ECs, both *in vitro* and in a murine model (Arenas et al., 2006; Roedel et al., 2002). Subsequent studies further indicate an impaired adhesion to be associated with a non-linear production of reactive oxygen species (ROS) in ECs, which is functionally based on a modulation of the transcription factor nuclear factor E2-related factor 2 (Nrf2) and anti-oxidative enzymes such as superoxide dismutase, catalase and glutathione peroxidase (Large et al., 2015). Further, clearance of apoptotic cells by macrophages is reported to result in non– or even anti-inflammatory responses (Gaipl et al., 2005; Voll et al., 1997). In line with that, a discontinuous appearance of apoptosis was observed in PBMCs following 0.1–0.3 Gy irradiation (Kern et al., 1999), which may foster a reduced adhesion. This is enhanced by a diminished surface expression of the adhesion molecule E-selectin on ECs (Hildebrandt et al., 2002; Roedel et al., 2002) or by the proteolytic cleavage of L-selectin from apoptotic PBMCs (Kern et al., 1999).

A key mechanism in the subsequent effector phase of inflammation covers the accumulation of monocytes and their differentiation into DCs and inflammatory macrophages (Valledor et al., 2010). The latter support the local inflammatory process by a variety of functions such as phagocytosis, presentation of antigens, cytotoxic activity and secretion of cytokines, ROS and nitric oxide (NO) (Fujiwara and Kobayashi, 2005). NO in turn impacts on vascular permeability, promotes tissue oedema and is involved in the development of inflammatory pain (Holthusen, 1997). Notably, irradiation at 0.3–1.25 Gy of activated macrophages resulted in decreased expression of the enzyme inducible nitric oxide synthase (iNOS) and NO production (Hildebrandt et al., 1998) in line with a hampered release of ROS and superoxide production (Schaue et al., 2002) that have mechanistically been linked to the anti-inflammatory and analgesic effects of low-dose exposure. In addition, irradiation with doses up to 2 Gy significantly decreased major histocompatibility (MHC) II expression and transmigration of macrophages (Wunderlich et al., 2019) but fostered chemotaxis of LPS activated macrophages without impacting viability and phagocytic functions (Wunderlich et al., 2015).

In order to establish experimental conditions mimicking as close as possible the patients situation, a human TNF-α transgenic mouse model was adopted to investigate the therapeutic and cellular effects of low dose irradiation. These animals overexpress the cytokine during their lifetime and develop a polyarthritis with joint swelling and deformation, synovial inflammation, cartilage damage, and bone erosion comparable to the human situation (Keffer et al., 1991). Irradiation of these mice with five times 0.5 Gy at early stages (4–6 weeks) revealed significantly reduced paw swelling and increased grip strength (Frey et al., 2009). More recent findings further indicate a significant reduction of inflammatory and erosive areas with a lowered detection of bone-resorbing osteoclasts and neutrophils. In addition, starting at a dose of 0.5 Gy, proliferation and expression of receptor activator of NF-kB ligand (RANK-L) in fibroblast-like synoviocytes decreased, numbers of differentiated osteoclasts and their bone resorbing activity also diminished in line with a discontinuous dose response relationship in osteoblast-induced mineralization (Deloch et al., 2018a). By contrast, irradiation with a single dose of 0.5 Gy displayed no harmful effects on cells in healthy joints (Deloch et al., 2018b). Thus, one can conclude that in this dose range irradiation may also act via osteo-immunological mechanism with the anti-inflammatory properties depending on the initial inflammatory status (Candeias and Testard, 2015; Frey et al., 2017). In conclusion, recent experimental *in vitro* data have characterized mechanisms and factors that may contribute to the anti-inflammatory effect of a low to medium dose exposure while *in vivo* models have confirmed improvements in a variety of clinical symptoms and parameters.

First clinical studies of patients that had been exposed to very low doses of high linear energy transfer (LET) radon gas (0.3 mSv) gave first hints that even here systemic immune modulations took place. Especially, activation status of immune cells was influenced as well as levels of TGF-β (Cucu et al., 2017; Kullmann et al., 2019; Ruhle et al., 2017). One of the most compelling observations in this patient population was a long-term shift within the CD4+ T cell compartment towards a higher proportion of circulating Tregs along with a fall in CD8+ T cells after cumulative doses of around 0.3 mSv (Cucu et al., 2017). Decreased levels of lymphocytes expressing the activation marker CD69 and a temporary rise in systemic TGF-β and higher IL-18 serum levels were found to directly relate to better pain control in patients after radon spa treatment (Kullmann et al., 2019).

#### 3.4.2. Lung injury during radiotherapy – A clinical example of radiation-induced pro-inflammatory pathology

The lung is one of the most radiosensitive organs, and RT-associated lung tissue inflammation can occur early (radiation pneumonitis, within 6 months) and late (chronic lung fibrosis, later than 6 months) after RT (Giridhar et al., 2015). Currently, radiation pneumonitis develops in about 30% of patients and is fatal in 2% (Palma et al., 2013). It is the main dose-limiting toxicity and can limit quality of life of cancer survivors. Genetic factors contribute to the severity of the radiation-induced pneumonitis and fibrosis (Gatti, 2001). Pathophysiology, molecular and cellular mechanisms of these injuries have been described (Giridhar et al., 2015; Huang et al., 2017), however still remain not fully understood.

After irradiation, as a consequence of radiation-induced oxidative stress the DDR is dysregulated in lung, resulting in a population of cells with unrepaired DNA damage. This in turn leads to destruction of alveolar epithelial and vascular ECs inducing tissue hypoxia. Oxidative stress and tissue hypoxia are important regulators of cellular signalling pathways controlling the production of various factors involved in the development of radiation-induced lung injury such as secretion of pro-inflammatory and pro-fibrogenic cytokines, growth factors and pro-teases that destroy the extracellular matrix and the barrier of lung tissue. Hypoxia-inducible factor 1α (HIF-1α) protein is an important transcription factor involved in the development of radiation-induced lung injury, which is expressed early after irradiation as a consequence of oxidative stress and tissue hypoxia and its expression progressively increases for months thereafter. Once induced, it regulates the expression of a series of genes involved in pathways like inflammation, oxidative stress response, proliferation, differentiation or angiogenesis (Imtiyaz and Simon, 2010; Jackson et al., 2012; Rabbani et al., 2010). Blood vessel dilation and increased permeability allows for accumulation of inflammatory cells (with macrophages being the most prominent cell types) at the site of irradiated tissue and in the alveolar cavity and cause a strong immune response. This acute inflammatory response may lead to radiation pneumonitis that induces a vicious circle of further inflammation and finally fibrosis induction. IR leads to release of cytokines as well. A first wave of cytokine release settles within two weeks. The second wave starts around 6–8 weeks post-irradiation (Rube et al., 2004) and is associated with accumulation of oxidative DNA damage, ROS or reactive nitrogen species (RNS), hypoxia, TGF-β expression, and decreased lung perfusion. TGF-β, in particular, TGF-β1, is mainly generated by activated macrophages and is a key player in development of fibrosis. It triggers proliferation and conversion of fibroblasts to myofibroblasts. Myofibroblasts in turn produce excessive collagen and other extracellular matrix components, and secrete angiotensin and hydrogen peroxide, which induce apoptosis in alveolar epithelial cells. Activin A is induced by TGF-β1 and it also stimulates production of collagen (Forrester et al., 2017). Fibrosis induces hypoxia and consequent release of pro-fibrotic and pro-angiogenic factors like TGF-β1 (Vujaskovic et al., 2001). These cycles are the basis of RT-induced chronic inflammatory lung disease.

Clinical predictors have been identified in an effort to minimize radiation pneumonitis. Since the inflammatory response underlies RT-related lung toxicities, immunological factors involved in this response may provide potential biomarkers to predict individual response to RT. Lierova et al. recently reviewed 56 articles that have evaluated the association between serum cytokines and pneumonitis in irradiated lung cancer patients, most of them receiving around 60 Gy (Lierova et al., 2018). Serum IL-6 and TGF-β1 tended to stand out in predicting susceptibility to subacute pneumonitis, whereas circulating IL-4 and IL-13 appeared more critical for delayed effects such as chronic inflammation and fibrosis. Higher grade pulmonary toxicity in lung cancer patients was associated with early changes during RT treatment in plasma levels of IP-10, MCP-1, eotaxin, IL-6, and tissue inhibitor of matrix metalloproteinase 1 (TIMP1) (Siva et al., 2016; Siva et al., 2014). The expression levels of follistatin, the member of TGF-β superfamily, fibrillin-2, which stores the latent form of TGF-β in extracellular matrix and inhibits its activation, and dermatopontin, which increases cellular response to TGF-β, predicted lung radiosensitivity manifested as radiation-induced fibrosis (Forrester et al., 2014). Vascular endothelial growth factor, E-selectin, I-selectin, basic fibroblast growth factor (bFGF), IL-1, IL-6, IL-8, IL-10, IL-12 and IL-18 have also been suggested as possible predictive biomarkers (Sprung et al., 2015).

Chronic inflammatory responses as predictors of radiation toxicity have also been described in patients treated with RT for other malignancies than lung cancer. A recent review by Marconi et al. included 15 publications from the last 5 years on breast cancer patients receiving RT, many with conventionally fractionated regimens to a total dose of 50 Gy. Some radiation-driven changes in cytokine levels persisted in these patients for months, such as IFN-γ and TGF-β. Similarly to lung, both IL-6 and TGF-β correlated with radiation toxicity, although tumour stage and timing were important factors, too (Marconi et al., 2019). Moreover, in 37 patients with prostate cancer receiving RT, cytokines were reported to appear in recurring waves with pro-inflammatory IL-1 and macrophage colony-stimulating factor (M-CSF) preceding TGF-β1 but more importantly the magnitude of these waves correlated with doses up to 9 Gy given in daily 1.8 Gy fractions (Kovacs et al., 2003).

Altogether, these clinical observations indicate that chronic inflammatory reactions are key players in the pathophysiology of high dose irradiation-induced normal tissue toxicity.

Although not directly linked to the topic of the paper, we would like to briefly mention that a cancer developing in an inflammatory microenvironment is in general more radioresistant compared to a cancer where chronic inflammation is not present. The link between local inflammation and cancer radioresistance is thoroughly investigated and several excellent reviews exist on this topic (Aggarwal et al., 2009; Jarosz-Biej et al., 2019; Multhoff and Radons, 2012; Woodward et al., 2010).

An important point to consider when comparing inflammatory responses after cancer RT with low dose radiation treatment of benign diseases is the different initial temporal and spatial states of the immune system. Many cancer patients have a more immune-suppressive tumor microenvironment, in contrast to the chronic inflammatory state in benign disease; both of which are far from the more balanced, neutral set-point in healthy people. As the baseline immune balance has shifted, the effect of radiation will shift also.

### 3.5. The role of genetic background in the variability of radiation effects on the immune system

Individual variation in response to IR is well documented in cancer patients receiving RT and radiosensitive patients can present therapy-related side effects such as fibrosis, necrosis, dermatitis, esophagitis, neuropathological disorders, inflammation and vascular damage, among others (Pavlopoulou et al., 2017). The mechanism of inflammation and fibrosis developing in lung after RT has been described in the previous chapter and it is evident that many of the adverse effects involve processes of the immune system. Heritability studies suggest that 58–82% of the individual variation in sensitivity to radiation and in the development of RT-related toxicity can be attributed to genetic factors (West and Barnett, 2011). Currently the role of the genetic background in individual radiosensitivity has almost exclusively been investigated for high doses and practically no data exist in the literature regarding low dose effects. We aim to provide a short summary of the data available regarding the role of the genetic background in the immune system changes influencing individual radiation response.

Several studies with murine models have shown genotype-dependent inflammatory-type responses after whole-body irradiation. For example, macrophages from CBA/Ca mice exhibited a pro-inflammatory phenotype, whereas those from C57BL/6 showed an anti-inflammatory phenotype after irradiation with 4 Gy (Coates et al., 2008; Rastogi et al., 2013). Moreover, different immune-related outputs were obtained after doses below 100 mGy in two mouse strains with different sensitivity to radiation-induced mammary carcinoma: the sensitive strain but not the resistant one showed transcriptional signatures of macrophage activation, pattern recognition receptor signalling, NK cell and TGF-β signalling. While changes in immune gene signatures were transient, activation of various cancer pathways in the sensitive strain after irradiation was a durable effect (Snijders et al., 2012). This would confirm the importance of genetic background in low dose IR-induced effects.

It is well known that rare mutations in DNA DSB repair genes can cause heritable syndromes, such as ataxia telangiectasia, that show extreme radiosensitivity. However, radiosensitivity is now considered not to rely only on a single rare gene mutation but to be an inherited complex trait that depends on the interaction of common risk alleles in a large number of genes (Andreassen et al., 2002). The different responses to RT in patients treated with the same radiation doses and not affected by one of the above-mentioned heritable syndromes, support the consensus of the involvement of common genetic factors in radiosensitivity (Guo et al., 2015).

The degree of the association between specific genes and pathways and sensitivity to radiation probably depends on the specific tissue involved and the particular endpoint analysed (Andreassen et al., 2002). Focusing on the immune system, some genetic variants (mainly single nucleotide polymorphisms (SNPs)) have been found to be associated with inflammation and fibrosis after radiation and in particular after RT (Table 4). For example, in early studies with relatively small sample sizes, polymorphisms in glutathione S-transferase alpha1 (GSTA1), glutathione S-transferase pi1 (GSTP1), TGF-β1, thioredoxin reductase 2 (TXNRD2), and X-ray repair cross-complementing 1 (XRCC1) genes were found to be significantly associated with radiation-induced fibrosis in breast cancer patients. On the other hand, polymorphisms in ataxia telangiectasia mutated (ATM), ERCC excision repair 5, endonuclease (ERCC5), mouse double minute 2 (MDM2), XRCC1 and XRCC3 were associated with fibrosis in naso-pharyngeal cancer patients. This would agree with the tissue-specificity of the associations in some cases. However, some studies failed to replicate the initial associations for TGF-β1, XRCC1 and XRCC3 (see Table 4 for references).

Later, more-powered studies were carried out (and some are still ongoing) in collaborative projects with thousands of patients, e.g. RadGenomics (Iwakawa et al., 2002), GENEPI (Baumann et al., 2003), Gene-PARE (Ho et al., 2006), RAPPER (Burnet et al., 2013), and REQUITE (Seibold et al., 2019; West et al., 2014). These collaborative projects have successfully identified association of alleles from the MHC III region with fibrosis in 2036 breast cancer patients and of ribonuclease L (RNASEL) gene with IL-6 levels in 2550 prostate cancer patients (Meyer et al., 2010; Talbot et al., 2012). However, a large study with 2782 patients reported no association of a SNP in TGF-β1 gene with RT-induced breast fibrosis (Barnett et al., 2012). Similarly, results from Zhu et al. with 2926 patients with several cancer types failed to find a correlation between TGF-β1 SNPs and fibrosis after RT (Zhu et al., 2013).

All these studies were focused on candidate genes suspected to be causally related to radiation toxicity, such as DNA damage recognition and repair, free radical scavenging or anti-inflammatory response. Besides the candidate-gene approach, few genome-wide association studies (GWAS) have been carried out to unravel genes important in radiosensitivity unbiasedly (Barnett et al., 2014; Fachal et al., 2014; Kerns et al., 2016; Kerns et al., 2010; Kerns et al., 2013a; Kerns et al., 2013b; Kerns et al., 2013c). Most of these GWAS provided evidence for associations of polymorphisms with overall toxicity after RT or with individual endpoints other than inflammation or fibrosis, so candidate gene approach studies previously mentioned (Table 3) could not be confirmed or refuted. However, some GWAS identified genetic variants that may underlie the variability of radiation effects on the immune system, specifically in prostate cancer patients treated with RT and who presented rectal bleeding, which could be caused by proctitis or inflammation of the rectum (Kerns et al., 2016; Kerns et al., 2013c). Recently, Kerns et al. performed a meta-analysis with 3871 prostate cancer patients and identified one SNP significantly associated with rectal bleeding (Kerns et al., 2020).

In conclusion the role of genetic background in shaping the individual immune response after high doses is well demonstrated even in the absence of identified consensus on the identity of the gene(s) or gene cluster(s) responsible for this effect. While studies investigating the role of genetic background in individual immune parameters to low dose IR are very scarce in the literature there is no doubt that an individual genetic signature is crucial in low dose response as well, especially taking into account the high inter-individual variability of low dose effects, which are most probably governed by genetic (and epigenetic) factors. This highlights the future need to focused research deciphering the role of genetic background in low dose regulated immune responses.

## 4. Conclusions

There is a wealth of scientific evidence based on epidemiological data from various cohorts exposed to different radiation doses, dose rates and radiation qualities as well as clinical studies and experimental *in vitro* and *in vivo* data, all collectively supporting the notion that low dose IR affects the immune system in multiple ways.

The most important of these findings can be summarized as follows:

1. Both studies on human subjects and experimental data indicate low dose radiation-induced damage on T cell immunity, especially CD4+ T cells. There is a fair amount of consensus in the literature on low dose radiation doses driving changes in the functional profile of CD4+ T cells, most often a shift towards a Th2 phenotype. CD8+ damage (both quantitative and functional) is also reported in most studies but there are discrepancies as to their radiosensitivity and their functional impairment depending on whether acute or long term chronic exposures were performed. It is also known that T cell activation can drive radioresistancy, which may explain some of these discrepancies.
2. Data regarding low dose radiation effects on humoral immunity mostly arise from epidemiological observations and relatively few experimental studies have focused on this component of the immune system. While most studies show some degree of humoral immune enhancement, these are in contradiction with sporadic reports on an increased proneness to bacterial infections among radiation exposed individuals, although this may vary with the infectious agent.
3. Within the innate immune system, NK cells are perhaps the best studied lineage and there is a relative consensus both in epidemiological and experimental data on the role of low dose radiation in stimulating certain types of NK cells. Reports about low dose radiation effects on other components of the innate immune system are either contradictory (DCs) or are too few (polymorphonuclear cells) to allow for any pertinent conclusion regarding their behaviour post exposure.
4. There is an interesting dichotomy when it comes to inflammatory responses in the context of low dose radiation. We can surely state that low-to-moderate doses of low and high LET radiation have anti-inflammatory effects on individuals with local inflammatory conditions and may result in improvement in a variety of clinical symptoms and parameters. On the other hand long-term observations of cohorts exposed to very low doses of radiation (acute or chronic) indicate a pro-inflammatory immune profile, which might contribute to increased incidence of chronic degenerative diseases with an inflammatory component.
5. Epidemiological studies were the first to raise the possibility of a radiation-induced acceleration of immune aging as well as radiation-induced metabolic perturbations in immune cells. Recently experimental data seem to validate these observations, though low dose studies in these topics are extremely few.

## 5. Recommendations for future research

Despite all the data presented here, there are still some research gaps that need to be filled and we present some recommendations for future studies:

1. Dysregulation of the immune system is thought to play a key role in many adverse health outcomes following exposure to IR. These can be cancerous or non-cancerous, such as cardiovascular, neuro-cognitive and metabolic diseases, as described by Tapio et al. in another paper of this special issue. Clearly one of the greatest gaps in epidemiological studies is that immune changes identified in different cohorts are not linked to changes in the incidence of particular diseases. These are intricately linked and need to be studied together, i.e. the role of the immune system in radiation-induced chronic non-cancer diseases such as cardiovascular, neuro-cognitive, metabolic diseases or autoimmune disorders. A better understanding of the mechanisms would allow us to develop effective countermeasures and to identify predictive biomarkers. Given the nature of the immune system, immune parameters considered to be in the normal range show very high variability in the population at large but are relatively constant at an individual level. Hence, looking at population means might easily mask individual changes. Ideally, in order to correctly evaluate individual changes they should be compared to pre-exposure values of the particular individual if possible, in addition to mean values of control population. Since most immune monitoring is performed on blood samples, future epidemiological studies should aim to include data from pre-exposure blood samples as well as include potential covariates/confounders such as additional stressors, lifestyle factors and genetic background.
2. Most evidence indicates that the relationship between IR exposure and immunological changes is anything but linear. In some instances, low, intermediate and high IR doses of different radiation qualities and applied in different dose rates induce discontinuous or biphasic effects on the immune system. However experimental data also show that low/intermediate versus high doses (or dose ranges) induce qualitatively different shifts in immune responses (e. g. the shift from Th1 to Th2 profile of T helper cells or a shift from a pro-inflammatory towards an anti-inflammatory response or vice versa). The effects of low/intermediate doses may depend on the initial inflammatory state of the irradiated tissue, the dose rate and quality of radiation. Big gaps exist in our knowledge of the molecular events responsible for these different response patterns. A better understanding of these mechanisms would enable a better control of radiation-induced immune changes. In line with that, one major characteristics of the immune system is a high degree of cellular motility and interaction dynamics of the different components to activate host-protective activities and to terminate immune responses to counteract chronic and harmful activation. Thus, multiple sampling and the usage of multiplexed, high-resolution *in situ* examination is recommended to unravel the complexity of immune interactions.
3. Syngenic animal studies and image guided irradiation of distinct locations in animals can greatly help in understanding radiation-induced immune changes. However, emphasis should be placed on long-term follow-up studies to identify persistent rather than transient immune changes. Currently available animal experiments are mostly investigating acute or short-term changes which are not really relevant for identifying late occurring pathological immune-related alterations.
4. It might be useful to take into account the state of the art in other fields in the study of the effects of IR on the immune system. Firstly, the immune system has a vast but limited number of mechanisms to respond to stressors. Commonalities of the mechanisms induced by stressors not related to IR can be expected and useful to elucidate radiation-related mechanisms. Secondly, immune responses in other organisms (including invertebrates and plants) may be one of the synergetic fields of research between radiobiologists and radioecologists. Third, as combined stressors reflect better the reality than exposure to IR alone, an exposome research approach considering the effects of environmental pollutants, nutrition, lifestyle factors as suggested above might be needed to reveal a more realistic effect of IR.
5. Concerning the medical applications of low dose IR, there is a need to further explore the immunomodulatory effect of both low and high LET radiation. For example for radon, the development of suitable *in vivo* platforms and basic models in line with extended translational and clinical research is seriously needed.

## Figures and Tables

**Table 1:** Doses and dose ranges and main type of studies related to the relevant dose ranges

	Low doses	Intermediate doses	High doses

Dose range	<100 mGy	100 mGy–1 Gy	>1 Gy
Studies^a^	Epidemiological Experimental	Epidemiological Clinical (LD-RT) Experimental	Clinical (RT) Experimental

a principal type of studies discussed in this review addressing this dose range.

RT: radiotherapy; LD-RT: low-dose radiotherapy.

**Table 2:** Overview of studies describing immunological changes in people exposed to different scenarios of irradiation.

Citation	Population/Site	Collective & Numbers	Dose	Endpoints	Remarks & Conclusions

Acute and chronic exposure					
(Ito et al., 2017)	A-bomb survivors	Cohort, 165 human tissue blocks from RERF archive	no (<5 mGy), low (5–200 mGy), moderate-to-high (<200 mGy) exposure; 11 unexposed controls	Immunohistology of thymus from pathology archive	Low dose is sufficient to result in decreased thymic function many years after exposure (years from exposure 9–41), accelerated thymus aging (involution)
(Kusunoki et al., 1998)	A-bomb survivors	Cohort, 159 exposed, 234 controls (<0.05 Gy)	1) <5 mGy; 2) >5 mGy to >1.5 Gy	PBMCs by flow cytometry, subsets of T, B, NK cells	A-bomb radiation might have triggered dominant Th2-cell responses, stimulating B-cell lymphopoiesis for a long period.
(Kusunoki et al., 2003)	A-bomb survivors	Cohort, 1280 individuals	2 groups: <5 mGy and >5 mGy	Subsets of CD4+ T cells	Memory CD4+ T-cells of individuals who received significant radiation doses in adulthood may have become dependent on a much less TCR Vβ families than unexposed
(Kusunoki et al., 2010)	A-bomb survivors	Cohort, 1035 individuals between 2006 and 2008	dose categories: <5 mGy, 5 mGy–0.5 Gy, 0.5 Gy–1.0 Gy, 1.0 Gy–4.0 Gy	PBMCs by flow cytometry, plasma TNF-alpha level (only a subgroup)	A-bomb survivors may have induced T-cell immunosenescence resulting in attenuation of T-cell-mediated immunity.
(Kyoizumi et al., 1992)	A-bomb survivors	203 A-bomb survivors, 6 Thorotrast patients, 18 thyroid disease patients; one Chernobyl person with high accidental exposure	A-bomb survivors: 1) <5 mGy (n = 125); 2) >1.5 Gy (n=78)	Mutation frequency of T-cell receptor (TCR) in PBMCs: flow cytometry with CD3 and CD4 antibodies assuming that mutant CD4+ T cells have only a small fraction of CD3 expression	No significant dose effects in A-bomb survivors.
(Kyoizumi et al., 2010)	A-bomb survivors	Cohort, 916 individuals	dose categories: <5 mGy, 5 mGy–0.5 Gy, 0.5 Gy–1.0 Gy, 1.0 Gy–4.0 Gy	PBMCs characterized by flow cytometry: subsets of memory T-cells by CD43 level	The steady state of the T-cell memory, which is regulated by cell activation and/or cell survival processes in subsets may have been perturbed by prior radiation exposure.
(Lustig et al., 2016)	A-bomb survivors	Cohort, 415 individuals, 2 time points: 55 and 66 years after exposure	3 exposure groups: 157 with no dose (<5 mGy), 123 with low (5 mGy–700 mGy), 135 with high (>700 mGy) dose	T cell counts, telomere length; serum cytokines, c-reactive protein (CRP)	Radiation damage drives changes in telomere length that persist in the progeny over half a century and therefore likely derived from the initial lesion. Radiation damage seems more severe in the young than the old. Telomere shortening likely cause functional defects that in the case of lymphocytes would lead to less T cell immunity and less myeloid function (less inflammatory cytokines).
(Yoshida et al., 2016)	A-bomb survivors	Cohort, 620 participants	dose range 0–1.736 Gy	PBMCs: telomere length of naïve and memory CD4+ T cells, total CD8+ T cells; metabolic status	Radiation exposure perturbs T-cell homeostasis involving telomere length maintenance by multiple biological mechanisms, depending on dose, and that long-term radiation-induced effects on the maintenance of T-cell telomeres may be modified by the subsequent metabolic conditions of individuals.
(Yoshida et al., 2019)	A-bomb survivors	Cohort, 14,349 participants	3 dose groups; <1 Gy (n = 1616), >1 Gy (n = 9393), control (not-in-town, n = 3340)	Longitudinal statistical analysis of blood cell counts	Radiation exposure might accelerate aging-associated clonal haematopoiesis, which could result in a long-lasting elevation of circulating monocytes.
(Ilienko et al., 2018)	Chernobyl	235 Chernobyl accident male clean-up workers exposed in 1986–1987; 45 matched non-exposed controls	Mean dose ± SD: 419.48 mSv ± 654.60; range 0.10–3,500 mSv	Lymphoctes: gene expression of candidate genes: BCL2, CDKN2A, CLSTN2, GSTM1, IFNG, IL1B, MCF2L, SERPINB9, STAT3, TERF1, TERF2,TERT, TNF, TP53, CCND1; relative telomere length; immune cell subsets, γ-H2AX and CyclinD1.	Cellular immunity, gene expression, telomere length, intracellular protein parameters are shown to be among perspective biological markers at a late period after radiation exposure.
(Kuzmenok et al., 2003)	Chernobyl	Chernobyl healthy clean up workers from Belarus: 134 workers and 89 matched controls	Dose estimation: 150 mGy–500mGy	PBMCs; isolated T-cells; mitogen stimulation	An approach to a more accurate analysis of the immunological disorders found after exposure to radiation from Chernobyl-related activities.
(Oradovskaia et al., 2011a)	Chernobyl	Liquidators, comparison of different time points (1986, 1987) and working conditions	Differences by time and timing of liquidation work	PBMC subpopulations; immunoglobulins	Specific features of changes in the immune system depend on dose of external gamma-irradiation. However, distinctions in the age dynamics of the immune system in liquidators in the presence and in the absence of cancer manifested themselves in a stable level of CD3+, CD4+, CD8(+)-T-lymphocytes, immune regulation index, CD95+, serum IgA at the age between 40 and 70 years.
(Saenko et al., 2000)	Chernobyl	57 liquidators, 21 controls	Physical dosimetry from official records; Chernobyl liquidators <0.25 Gy	erythrocyte variant cells bearing a mutated glycophorin A (GPA) surface marker	In Chernobyl clean-up workers the TCR mutant frequency was significantly higher than in control non-irradiated individuals.
(Chang et al., 1999a)	Home environment, Taiwan	196 exposed residents with 2–13 years of exposure in their homes; 55 close relatives non-exposed	Protracted gamma-radiation, mean excess cumulative dose: 169 +/− 272 mSv; mean annual excess dose 24+/− 29.9 mSv	blood: lymphocyte subpopulations	Significant immunological effects were observed in those who received chronic low-dose radiation exposure.
(Jain and Das, 2017)	Kerala, India	Cohort, 36 healthy male individuals, age 28–52 living in different level natural background radiation areas	5 dose groups based on annual background dose received; I (control): <1.5 mGy/year; II: 1.51–5.0 mGy/year; III: 5.01–15.0 mGy/year; IV: >15 mGy/year; individual dosimetry	Gene expression in PBMCs, gene ontology, pathway analysis	Individuals exposed to background doses of >5 mGy/year showed alterations in the expression of genes involved in immune system-related pathways.
(Takahashi et al., 1999)	Marshall Islands	Cohort, 4766 individuals aged to be at risk from exposure of radioactive fallout by the US nuclear testing programme on Bikini and Eniertah atoll (1946 and 1958)	No dose estimation provided	Thyroid examination by ultrasound, thyroid hormone determination, anti-thyroid antibodies, questionnaire, iodine status (urine samples)	Dietary intake of iodine needs to be taken into account when looking at the link between radiation exposure and thyroid nodules.
(Attar et al., 2007)	Ramsar, Iran	100 individuals from villages with high level natural background radiation (HLNBR) and villages with low background radiation	13 times higher than normal in HLNBR area	PBMCs for functional assays, cytokines IL-2, IL-4, IL-10, IFN-gamma	Immune system adaptation in individuals living in high natural radiation background areas
(Borzoueisileh et al., 2013)	Ramsar, Iran	50 individuals aged 25–35 years, exposure duration 10–35 years, different level natural background radiation areas	estimated dose of 10.2–260 mSv/year in Ramsar area	Flow cytometry of PBMC subpopulations: CD4+/CD45+ (T-helper-cells), CD8+ (cytotoxic T-cells), NK cells and CD107a-cells	Multiple immune system alterations
(Ghiassi-nejad et al., 2002)	Ramsar, Iran	Individuals from HLNBR areas vs normal background radiation area	Annual radiation absorbed dose from background radiation up to 260 mSv/year	chromosome aberrations after *in vitro* challenge dose with 1.5 Gy	An adative response in terms of chromosomal aberrations induced by chronic low dose exposure
(Ghiassi-Nejad et al., 2004)	Ramsar, Iran	50 exposed individuals from HLNBR area aged 40+/−16 years; 30 matched controls	Estimated annual effective dose: 1.6–42 mSv/year; 2.3 mSv/year for controls	Immunoglobulins IgM, IgG, IgA, IgE, complement (C3, C4, C1-inactivator), rheumatoid factor, CRP; flow cytometry of PHA stimulated and unstimulated PBMCs with CD3, CD4, CD5, CD69 markers; cytogenetic analysis	Stimulation of Th2 response is discussed
(Molaie et al., 2012)	Ramsar, Iran	Subjects from high and low level natural background radiation areas	high and low natural background radiation	Neutrophil chemotaxis, Nitro-Blue Tetrazolium (NBT), antioxidant effects, cytokines (IL-2, IL-4) levels	The level of IL-4 increased in individuals who lived in area with high levels of natural radiation, which could lead to Th2 pattern of immune response
(Akleyev et al., 2019)	Techa River, Mayak area	Cohort, 66 residents of the Techa River basin contaminated due to release of liquid radioactive waste from the Mayak Production Association (Plutonium) in 1952; groups: 29 people with vs 37 people without increased TCR-mutations	Dose estimation according to the Techa River Dosimetry system 2009 (TRDS-2009): main group (TCR-mutations): dose rate to bone marrow 0.21+/− 0.02 Gy/year 1951, absorbed dose = 0.89+/−0.09 Gy (individual 0.09–1.96 Gy) comparison group: dose rate to BM 0.25+/−0.02 Gy/year 1951; absorbed dose = 1.03 +/− 0.07 Gy (range 0.03–2.34 Gy)	Number of CD19+, CD3+, CD3+CD4+, CD3+CD8+, CD3+CD4+/CD3+CD8+ cell ratio, immunglobulins (IgA, IgM, IgG); number of neutrophils, monocytes and their phagocytotic, lysosomal activity and intensity of intracellular oxygen-dependent metabolism; eosinophils, basophils, CD16+CD56+ and CD3+CD16+CD56+ lymphocytes; cytokines; colony stimulating factors: GM-CSF, G-CSF, TNF-alpha; IFN-alpha, IFN-gamma lymphocyte subsets; 30 cytokines	Low dose exposure induced long term changes of the innate immune system; immune system seems to react to DNA damage driving innate immune cell activation in an effort to eliminate TCR-mutated lymphocytes other than by apoptosis
(Li et al., 2019)	Yangjiang district, China	100 women exposed to HLNBR, 100 matched controls	estimated cumulative dose in exposed group: 58.5–249.13 mSv		Immune function was found to be affected in humans exposed to long-term low dose radiation: increase in CD8+ T-cell numbers and upregulated inflammatory biomarkers like IFN-gamma, MCP-1, sIL6R, EGFR, CRP
(Gyuleva et al., 2015a)	Nuclear power plant workers	Nuclear Power Plant (NPP) ‘Kozloduy”, Bulgaria. 438 persons working in NPP; 10 year survey	Cumulative doses between 0.06 mSv and 766.36 mSv and a control group with 65 persons	Flow cytometry of lymphocyte subpopulations, serum levels of IgG, IgA, IgM	Assumption that while the adaptation processes are dominated with low prevalence of T-helper 1 (Th1) immune response to cumulative doses <100 mSv, a switch to TH-2 response occured at doses >100 mSv.
(Gyuleva et al., 2015b)	Nuclear power plant workers	NPP “Kozloduy”, Bulgaria. 438 persons working in NPP; 10 year survey	Cumulative doses between 0.06 mSv and 766.36 mSv and a control group with 65 persons;	Flow cytometry measurements of T, B, natural killer (NK) and natural killer T (NKT) cells	Some of the studied parameters could be interpreted in terms of adaptation processes at low doses. At doses above 100–200 mSv, compensatory mechanisms might be involved to balance deviations in lymphocyte subsets. Some observed variations in some cases on the immune system might be due to other unknown factors.
(Gyuleva et al., 2018)	Nuclear power plant workers	NPP “Kozloduy”, Bulgaria. 105 employees	control, 4 dose groups: <25 mSv; <100 mSv; <200 mSv; > 200 mSv	lymphocyte subpopulations; serum IgG, IgM, IgA; IL-2, IL-4, IFN-gamma	The observed even slight trends in some lymphocyte populations and in cytokines profile allow to assume a possibility of a gradual polarization of Th1 to Th2 immune response at dose range 100 to 200 mSv.
(Rees et al., 2004)	NPP workers	British Nuclear Fuels, Sellafield: 194 male radiation workers >200 mSv (mean 331.5 mSv); 131 workers <27.5 mSv (mean 13.9 mSv)	Film badge dosimetry over 30.6 years vs 23.9 years; cumulative exposure >200 mSv vs <27.5 mSv	PBMCs: T cell and B cell subsets	No significant immunological effects in male radiation workers at >200 mSv compared to <27.5 mSv; smoking is an important confounding variable.
(Ahmad et al., 2016)	Radiology workers	60 healthy individuals working in different medical diagnostic units: 20 exposed, 40 matched controls	mean dose: 2.03 mSv/year; duration of radiation exposure: 16 years	Superoxide, DNA oxidation, cytokines	The data suggest a pro-inflammatory response at doses above 17 mSv. A threshold and non-linearity is discussed.
(Godekmerdan et al., 2004)	Radiology workers	50 radiology workers vs 35 age-matched healthy controls, mean age 30.1 +/− 7 vs 31.5 +/−5.8 years; 48% vs 0% smokers;	<3.5 mSv/year for 86%; the rest received above that; exposure time >5 years in 48%	Subgroups of PBMCs; serum complement and Igs	T helper cell and humoral immune components are compromised.
(Karimi et al., 2017)	Radiology workers	30 radiology workers vs 20 control laboratory workers	Exposure <50 mSv	PBMCs, PHA stimulation assay; serum cytokines	No dose response tested. A shift towards Th1 responses by low dose radiation is discussed.
(Klucinski et al., 2014)	Radiology workers	X-ray diagnostics units: 47 workers (14 men, 33 women); control group 38 (10 men, 28 women) non-exposed	Period of employment: 1–33 years with annual effective dose < 1 mSv	Flow cytometry of B-cell subsets: B-cells (CD19 +), B1-cells (CD5+ CD19+), memory B-cells (CD27+ CD19+)	Association of suppressive influence of low level ionizing radiation on B and memory B-cells is discussed.
(Rybkina et al., 2014)	Mayak production workers	Mayak Production Association workers cohort; 91 workers and 43 controls	14 workers exposed to external gamma-rays (total dose 05–3.0 Gy), 77 workers with combined exposure (external gamma-rays and internal alpha radiation from incorporated plutonium)	Cytokines: TGF-beta1, TNF-alpha, IFN-gamma, IL-1beta, IL-8; immunoglobulins: IgM, IgG, IgA, IgE; p53, HSP70, MMP-9; lymphocyte subsets	Chronic occupational IR exposure of workers induced a depletion of immune cells in peripheral blood
(Zakeri et al., 2010)	Interventional cardiologists	37 interventional cardiologist vs 37 control;	8.14 mSv/year (range 1.2–27.8) for 12.1 +/− 6.6 years and an accumulated dose over the last 5 years of 30.5 +/− 24.3 mSv	serum cytokines and Igs; cytokine release from activated lymphocytes, PBMC phenotypes	No dose response observed due to low case numbers
Studies on radiation-exposed children					
(Imaizumi et al., 2008)	A-bomb survivors	A-bomb survivors exposed in utero; 328 persons (mean age 55.2 year; 162 male); examination 55–58 year after exposure in utero	mean maternal uterine radiation dose 0.256 Gy; <5 mGy, 5 mGy–0.1 Gy, 0.1–0.5 Gy, 0.5–1 Gy, > 1 Gy	Thyroid: solid thyroid nodules and cysts; blood: antithyroid antibodies (ATAs): antithyroperoxidase (TPO-Ab) and antithyroglobuline (TgAb)	Antithyroid antibodies were not associated with dose or gestational week at exposure. No significant dose–response relationship for autoimmune thyroid disease in the in utero-exposed subjects (similar to exposed children).
(Chang et al., 1999)	Home environment, Taiwan	289 children exposed at kindergarden in 1983–92 to continuous low dose Co-60 gamma irradiation vs 751 aged- and sex-matched exposed to lower dose, studied 5–7 years later	High dose group estimated 21–85 mSv in total (200–800 chest X-rays) compared to low dose group 2–5 mSv (20–50 chest X-rays)	Blood draw for basic differential blood counts	Persistent changes in haematopoietic system following chronic low dose expsoures in the observed children.
(Agate et al., 2008)	Chernobyl	Cohort, 1433 sera from adolescents 13–17 years (born 1982–1986); additional 1441 control sera from aged-matched and sex-matched children in Denmark and Sardinia	Contaminated areas included Klintsy (Russia), Korosten (Ukraine) and Lelchitsky (Belarus) at 555–1480 kBq/m2; iodine deficiency prevalent in both contaminated and non-contamined areas;	ATAs: TPO-Ab, TgAb; thyroid function based on circulating levels of thyroid-stimulating hormone (TSH) and free triiodothyronine (FT3) and thyroxine (FT4)	TPO-AB prevalence in adolescents exposed to radioactive fallout was still increased in Belarus 13–15 years but a lot less than at 6–8 years after the Chernobyl accident but normal thyorid function possibly suggests a transient radiation-induced autoimmune reaction without triggering clinical thyorid autoimmune disease.
(Chernyshov et al., 1997)	Chernobyl	120 children aged 6–13 years from 15 radiation-contaminated areas in North Ukraine after Chernobyl accident with/withour recurrent respiratory disease (RRDC); 87 children from non-contaminated areas with/without RRDC	Exposed children from areas within a 40–75 km radius from the reactor; estimated dose of Cs-137 and Sr-90 of 0.57–3.09 mSv over 3 years; two groups < or >1 mSv	Major lymphocyte subsets analysed in whole blood by flow cytometry	Long-time exposure to low radiation doses may affect the immune balance, especially in vulnerable populations.
(Kasatkina et al., 1997)	Chernobyl	89 children from Uritzky region (416 km north of Chernobyl); 116 non-contaminated Kolpnyansky area; 2 age groups: age at exposure in utero (n = 89 and n = 100 controls) or 8–9 years (n = 81 and n = 97 controls)	Average Cs-137 soil contamination 1.71 Ci/km2 (range 0.18–3.97)	Thyroid dimension by clinical exam and ultrasound; thyroid function (hormones); autoantibodies; fine needle aspiration	Autoimmune thyroid disease markedly increased in children with poor iodine nutrition who were exposed to low level radiation. Low level radiation may induce thyroid gland changes in children who had inadequate iodine intake.
(Pacini et al., 1998)	Chernobyl	472 patients with thyroid carcinoma from Belarus diagnosed at <21 year compared to aged-matched controls with thyroid carcinoma from Italy and France: a) <14 year children (n = 372); b) adolescent 14–21 year (n = 100);	Radioactive contamination I-131 in Belarus: ranging from 185 to 37,000 kBq/m2	Thyroid immunity and function: T4, T3, TSH, thyroid ATAs: TPO-Ab, TgAb	Young children (<5 year) are especially vulnerable to radiation-induced thyroid cancer that tend to be more aggressive in nature and associated with signs of thyroid autoimmunity
(Sheikh Sajjadieh et al., 2012)	Chernobyl	Chernobyl area: children aged 4–18 years with/without diagnosed irritable bowel disease	Internal whole body radioactivity due to Cs-137; group1 (21 children aged 4–9): 1.9 Bq, group2 (26 children aged 10–13): 1.85 Bq, group3 (28 children aged 14–18): 2.01 Bq, group4 (21 healthy childen aged 5–15): 1.8 Bq	Lymphocyte subsets, cytokines: IL-4, IFN-gamma	Children with irritable bowel disease had less CD4+ T-cells, a higher level of IL-4 and a lower level of IFN gamma, suggesting a stronger polarization toward a Th2 phenotype. There was no difference with age, suggesting that there was no radiation-dose effect.
(Vykhovanets et al., 2000)	Chernobyl	6–14 year old children in radiation-contaminated areas in North Ukraine after Chernobyl accident (n = 78; 5 years after accident) and 141 different children (8–10 years after accident); children with recurrent respiratory disease (RRDC, mean age 8.3 years) vs non-RRDC in contaminated areas; n = 61 (1991) and n = 87 aged-matched controls from non-contaminated areas	Low doses of radiation to the whole body from Cs-137 ranging from 1.79 to 53.7 mSv (1991) and 2.17–29.33 mSv (1994–96) and various doses of radiation to the thyroid from I-131 as fallout	Major lymphocyte subsets analysed in whole blood by flow cytometry	Possibility that long-term exposure to low doses of Cs-137 may have altered the immune balance in especially vulnerable children. The shifts in circulating lymphocyte subsets between healthy children and those with RRDC may be attributed to long-term low-dose exposure of the whole body to radiation from Cs-137 and exposure of the thyroid to radiation from I-131.

**Table 3:** Summary of described immune effects by radiation exposure.

Radiation effect	Possible immune marker

Imbalance of peripheral blood mononuclear cells	Changes in B cell count Changes in T cell count Changes in T cell subpopulations Changes in NKT count
Acceleration of immunoaging	Reduction of naive T cells Expansion of memory T cells Thymus involution Reduction of telomere length of leukocytes
Humoral immune response	Changes in immunoglobulin level (IgA, IgM, IgG, IgE)
Inflammation	Pro-inflammatory response by cytokines

**Table 4:** List of genes reported to be significantly associated with immune-associated endpoints after radiotherapy. Only studies with >100 samples were included.

Endpoint	Tumor type*	Genes associated with the endpoint	Genes with contradictory results	References

Dermatitis	BC	*ABCA1, IL12RB2*		(Isomura et al., 2008)
	HNC	*GSK3B, MDM2, XRCC1*		(Borchiellini et al., 2017; Chen et al., 2017; Yu et al., 2016)
Esophagitis	LC	*BLM HSPB1, PRKCE, TGFB1, TNFSF7*		(Guerra et al., 2012; Lopez Guerra et al., 2011; Pu et al., 2014; Zhao et al., 2016)
Fibrosis	BC	*ATM* (lung fibrosis), Class III MHC region, *GSTA1, GSTP1, TXNRD2*	*TGFB1, XRCC1*	(Barnett et al., 2010; Barnett et al., 2012; Edvardsen et al., 2013; Edvardsen et al., 2007; Giotopoulos et al., 2007; Grossberg et al., 2018; Quarmby et al., 2003; Seibold et al., 2015; Talbot et al., 2012; Terrazzino et al., 2012)
	HNC	*ATM, ERCC5, HDM2, XRCC1*	*XRCC3*	(Alsbeih et al., 2014; Borchiellini et al., 2017; Cheuk et al., 2014)
	Several cancers	*ATM*		(Zhang et al., 2016)
IL6 levels	PC	*RNASEL*		(Meyer et al., 2010)
Mucositis	HNC	*APC, ATG10, ATG16L2, EDN1, NBN*	*XRCC1*	(Chen et al., 2017; Ma et al., 2017; Pratesi et al., 2011; Venkatesh et al., 2014; Yang and Liu, 2019; Yu et al., 2016)
Pneumonitis	LC	*AKT2, ATG16L2, BMP2, DDX58, ERCC1, GSTP1, HIPK2, IL4, IL8, LIN28B, MTHFR, MUS81, NEIL1, NFKBIA, PI3CA, RAD51, SP-D, TNF, TOPBP1, TP53*	*APEX, ATM, CBLB, HSPB1, IL1A, IL13, LIG4, MIF, NOS3, TGFB1, TNFRSF1B, VEGF, XRCC1*	(Chen et al., 2013; Du et al., 2018a; Du et al., 2018b; Hildebrandt et al., 2010; Li et al., 2014; Li et al., 2016; Mak et al., 2012; Niu et al., 2012; Pang et al., 2013; Pu et al., 2014; Tang et al., 2016; Tang et al., 2019; Tang et al., 2020; Voets et al., 2012; Wang and Bi, 2010; Wen et al., 2018; Wen et al., 2014; Xiong et al., 2013; Xu et al., 2019; Yang et al., 2017; Yang et al., 2011; Yin et al., 2011a; Yin et al., 2011b; Yin et al., 2012a; Yin et al., 2012b; Yuan et al., 2009; Zhang et al., 2010; Zhao et al., 2016)

* BC, breast cancer; HNC, head and neck cancer; LC, lung cancer; PC, prostate cancer.
